# Supplementary material for: Facile Preparation of Gold-Decorated Fe3O4 Nanoparticles for CT and MR Dual-Modal Imaging
Source: Int J Mol Sci. 2018 Dec 14;19(12):4049. doi: 10.3390/ijms19124049 (PMC6321430; doi:10.3390/ijms19124049)
Supplement: Supplementary file 1 [file ijms-19-04049-s001.pdf]

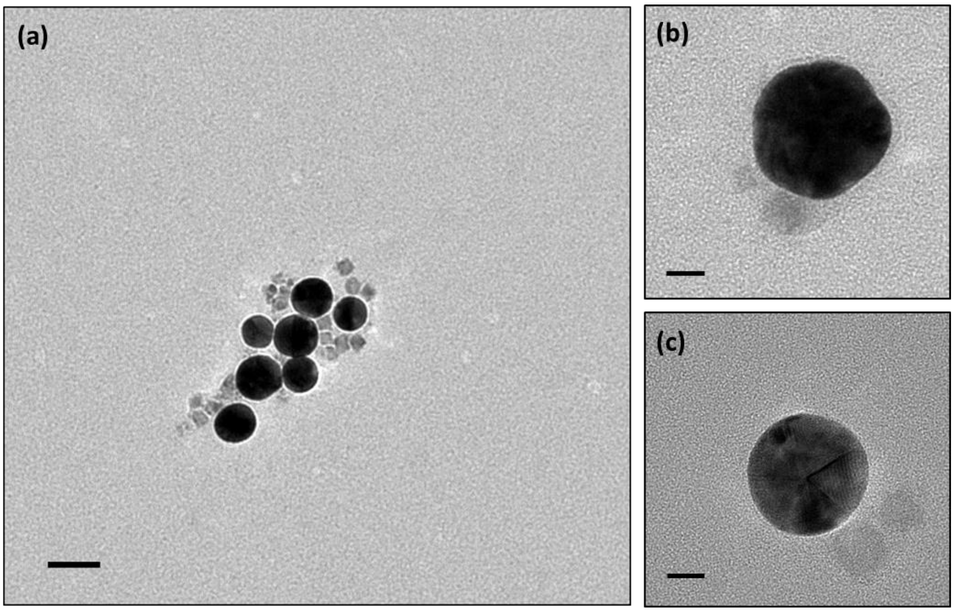

Figure S1. (a) TEM image of Au-Fe_3O_4 nanocomposites. The sizes of Fe_3O_4 and Au nanoparticles are around 10 nm and 30 nm, respectively; (b,c) TEM images of a single Au-Fe_3O_4 nanocomposite; The scale bars are 50 nm, 10 nm and 10 nm in (a-c), respectively.
